# Material utilization of green waste: a review on potential valorization methods

**DOI:** 10.1186/s40643-021-00367-5

**Published:** 2021-02-22

**Authors:** Alexander Langsdorf, Marianne Volkmar, Dirk Holtmann, Roland Ulber

**Affiliations:** 1 Institute of Bioprocess Engineering and Pharmaceutical Technology, University of Applied Sciences Mittelhessen, Wiesenstrasse 14, 35390 Giessen, Germany; 2 Institute of Bioprocess Engineering, University of Kaiserslautern, Gottlieb-Daimler-Strasse 49, 67663 Kaiserslautern, Germany

**Keywords:** Green waste, Biomass pretreatment, Biomass conversion, Valorization, Carbonization

## Abstract

Considering global developments like climate change and the depletion of fossil resources, the use of new and sustainable feedstocks such as lignocellulosic biomass becomes inevitable. Green waste comprises heterogeneous lignocellulosic biomass with low lignin content, which does not stem from agricultural processes or purposeful cultivation and therefore mainly arises in urban areas. So far, the majority of green waste is being composted or serves as feedstock for energy production. Here, the hitherto untapped potential of green waste for material utilization instead of conventional recycling is reviewed. Green waste is a promising starting material for the direct extraction of valuable compounds, the chemical and fermentative conversion into basic chemicals as well as the manufacturing of functional materials like electrodes for electro-biotechnological applications through carbonization. This review serves as a solid foundation for further work on the valorization of green waste.
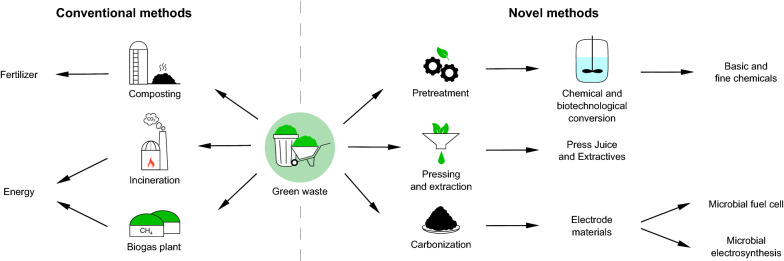

## Introduction

Especially in urban areas, various waste material streams occur, which are recycled economically unprofitable until now. Concerning the increasing interest in circular economy as well as bioeconomy, it is desirable that all waste is recycled as profitably and sustainably as possible. This is particularly important considering the intensified climate change as well as an increasing world population and urbanization. According to the updated bioeconomy strategy from the European Commission, EU cities should become a major hub for circular bioeconomy to exploit urban bio-waste (European Commission 2018). Due to the depletion of fossil resources and pressing environmental issues, lignocellulosic biomass is increasing in attractiveness as a feedstock, not only for the energy sector but also for the chemical industry. In 2015, the manufacture of bio-based chemicals, excluding biofuels, recorded the highest value-added annual growth with + 26% (European Commission 2018). One of the major biomass waste streams occurring in urban areas, which is not utilized economically worthwhile, is green waste. For example, in the Berlin (Germany) city districts, over 120,000 t a^−1^ of herbaceous fresh matter accumulate, comprising 72,000 t a^−1^ of leaves from yards and roads and 48,000 t a^−1^ of grass cuttings from lawns, roadside greenery, and biotope areas (Medick et al. 2017). With increasing urbanization, the number of urban green spaces increases. Subsequently, the amount of green waste is growing, affecting city development and the environment (Zhang et al. 2013).

Within the current literature, there is no uniform definition for the term green waste. Typically, green waste includes biodegradable garden waste and public park waste (Eades et al. 2020). Garden or park waste is generated by the maintenance of private gardens or public parks and can consist of organic materials like grass clippings, hedge cuttings, pruning, leaves, and wood as well as inorganic materials like stones and soil (Boldrin and Christensen 2010). According to the European Waste Catalogue, this garden and park waste also includes cemetery waste (German Federal Statistical Office 2020). Green waste is also defined as the biodegradable fraction of municipal solid waste (MSW) (Zhang and Sun 2016). Most of the time, the components of green waste are described as grass, leaves, and branch cuttings (Inghels et al. 2016; Zhang et al. 2013; Zhang and Sun 2016, 2014). Leaves and grass cuttings, without woody materials, are also referred to as herbaceous green waste (Medick et al. 2017). Herein, green waste is defined as grass and leaves collected from public parks, private gardens as well as cuttings from roadside greenery including a small amount of branches or other woody materials, resulting in a mainly non-woody or low-lignin and herbaceous green material.

Nowadays, composting and the subsequent application as fertilizer in agriculture is the method of choice for the majority of current green waste management in and around the largest cities in Germany (e. g. Berlin) (Medick et al. 2017). Another uprising method is the production of biogas. According to the German Federal Statistical Office (German Federal Statistical Office 2020), about 5.6 million tonnes of biodegradable garden and park waste were produced in 2018 within Germany. Almost the entire amount (5.5 million tonnes) was recycled. In 2017, there were 631 green waste composting plants for exclusively green waste and 297 fermentation plants (biogas plants including combined fermentation and composting plants) throughout Germany (German Federal Ministry for the Environment, Nature Conservation and Nuclear Safety 2019). These plants produced 4.2 million tonnes of compost, almost 3.4 million tonnes of digestate, and 626 million m^3^ of biogas in 2017 (German Federal Environment Agency 2020). The fermentation residues of biogas plants also represent a valuable fertilizer and humus supplier for application in agriculture. A lot of lignocellulosic material around the world is also disposed of by burning. However, the burning of waste biomass releases particulate matter, polycyclic aromatic hydrocarbons, dioxins, and toxic air pollutants, which can affect air quality negatively especially in urban areas (Sivertsen 2006). On top of that, its high amount of moisture, oxygen, and alkaline earth metals, as well as the production of flue gas emissions, make green waste unattractive for incineration (Shao et al. 2019). Besides the mentioned common methods, the production of ethanol is also an interesting and one of the most economically feasible methods for recycling of lignocellulosic biomass (Pérez et al. 2002). There is a large number of publications on bioethanol production from grass and even more from biomass in general. Due to the structural features of lignin, plants with low lignin and high cellulose/hemicellulose content are increasingly interesting for biofuel production. For example, Mohapatra et al. reviewed different methods of bioethanol production from herbaceous biomasses like *Miscanthus*, switchgrass, Napier grass, or Coastal Bermuda grass (Mohapatra et al. 2017). Nevertheless, composting is generally the method of choice for green waste utilization based on economic and environmental aspects (Zhang et al. 2013; Zhang and Sun 2014). Altogether the recycling of green waste still costs considerably more than thereby is generated.

Nowadays, there are plenty of publications regarding the utilization of woody biomass with low moisture content and/or high lignin content as well as agricultural residues for the generation of higher value, while the utilization of herbaceous material has been researched comparatively less. Regarding herbaceous or green biomass, energy crops like *Miscanthus*, switchgrass, *Arundo donax*, *Populus nigra*, or *Eucalyptus camaldulensis* (Ventorino et al. 2017) have been a focus of research, since the production of biofuels and bioenergy is currently a popular research topic. However, such energy crops hold the disadvantage of competing with food for bio-productive land (Johansson and Azar 2007). With an increasing world population, the food–fuel competition will become a more pressing issue. A great advantage of green waste compared to energy crops is that it does not require any arable land but arises anyway. Therefore, the feedstock production costs are zero, if you do not consider the cultivation, the care of plants and lawns, or the collection and pretreatment of green waste. The greater difficulty in processing green waste lies in the collection, since it does not accumulate in certain fields, but is distributed over large areas. In metropolitan regions, green waste is typically collected and disposed of by public waste management companies, park departments, and private landscaping/gardening companies, while in rural areas green waste is collected and disposed of by municipal green waste collection points (Medick et al. 2017). Furthermore, the processing of green waste cannot be optimized by genetic or molecular modifications of the feedstock. The probably largest problem with the utilization of green waste is its heterogeneity. The heterogeneity of green waste from a single meadow, lawn, park, or garden is manageable. When green waste is collected from a large number of sources, there is a huge incline in heterogeneity. In addition, green waste composition varies depending on several other factors, which are explained more in detail hereinafter. Unfortunately, the publications investigating the composition and utilization of green waste in its entirety are limited. Therefore, in this review, especially low-lignin or non-woody herbaceous materials that might appear in green waste, predominantly grasses, but also leaves or low-lignin branches, should be considered for the generation of higher value. Agricultural residues like wheat straw or corn residues are left out since there are already plenty of publications investigating these bio-wastes. Furthermore, the potential energetic use of biomass, including methane and ethanol production, will not be considered in this review for the same reason. Instead, the current state of science for the material utilization of herbaceous materials and green waste is presented to lay a foundation for future work on green waste valorization.

## Green waste composition

The heterogeneity of green waste is caused by various factors. First of all, the type of plants arising in green waste varies depending on the place in which the green waste was collected. In Singapore for example, abundant green waste materials are dead eucalyptus leaves (Liu et al. 2013), which do hardly ever occur in European cities. Zhao et al. investigated the plant species composition in built-up areas of Beijing City and determined a total of 618 plant species within different green spaces (Zhao et al. 2010), which underlines the possible variety of plants in green cuttings, even in dense urban areas. Differences in composition and physical shapes, also through seasonal variations, result in challenges in transportation and storage (Liu et al. 2013). A pretreatment at the place of collection of green waste might be advantageous for further logistics. While public park waste is relatively easy to collect separately by authorities, garden waste from private households is rarely separated from other bio-waste (Eades et al. 2020). Since garden and kitchen waste are often combined into organic waste, it is difficult to determine exact numbers for the green waste of private households. Nevertheless, some studies can be found on the composition of MSW from individual cities, regions, or countries. The amount of garden-related grass and wood waste from MSW varies mainly due to residential buildings being equipped or lacking private gardens (Gu et al. 2015). Hanc et al. described the differences of bio-waste from an urban settlement (multi-story apartment buildings) and individual family houses with small gardens in Prague, Czech Republic during different seasons (Hanc et al. 2011). While urban bio-waste varies hardly, bio-waste from family houses is influenced by the seasonal garden activities, since over 75% of the average yearly bio-waste from family houses consists of grass, leaves, wood, and plants (Hanc et al. 2011). The strong seasonal variations of green waste have also been shown by Boldrin and Christensen (Boldrin and Christensen 2010). They showed that the amount of garden waste in Aarhus, Denmark varies from 19.4 kg person^−1^ month^−1^ in the summer to 2.5 kg person^−1^ month^−1^ in winter. In the study, garden waste from the summer consisted of more than 90% of “small stuff” like grass clippings, flowers, hedge cuttings, or soil, while the winter was dominated by woody materials (Boldrin and Christensen 2010).

Besides the regional and seasonal variation in green waste composition, the physicochemical characteristics of the individual plants vary depending on several factors. The composition of herbaceous materials includes carbohydrate polymers like cellulose and hemicellulose, phenolic polymers (lignin), and other components like proteins, acids, salts, and minerals (Mohapatra et al. 2017). The chemical composition of a plant differs depending on the species, its developmental stage (maturity), and the environmental conditions in which it was grown (Mohapatra et al. 2017). Herrmann et al. showed the differences in dry matter, crude fat, crude protein, crude fiber, and sugar of freshly harvested samples from three typical grassland biotopes from north Germany depending on the type of grassland and date of harvest (Herrmann et al. 2014). Differences in the concentrations of cellulose, hemicellulose, lignin, fibers, and crude proteins depending on the harvest date have also been demonstrated by Mashingo et al. (Mashingo et al. 2008). Kim et al. displayed the effect of growing conditions on prairie cordgrass (*Spartina pectinata L.*), resulting in significant differences in the composition of the plant between two years (Kim et al. 2015). Typical protein contents of herbaceous material range from 1.5 to 4.5% for grassland (Oregon, USA) (Juneja et al. 2011), 5 to 6% in elephant grass (Menegol et al. 2016; Scholl et al. 2015), 12.3% in timothy (Alvo and Belkacemi 1997) to 18.5% in alfalfa (Alvo and Belkacemi 1997). The amount of extractives can differ similarly as shown for e. g. *Miscanthus* (6.9%) and switchgrass (13.6%) (Reza et al. 2013). The concentration of fats/lipids was shown to be around 2.5% of dry weight for yard waste as well as grass and leaves (Komilis and Ham 2003).

**Fig. 1:**
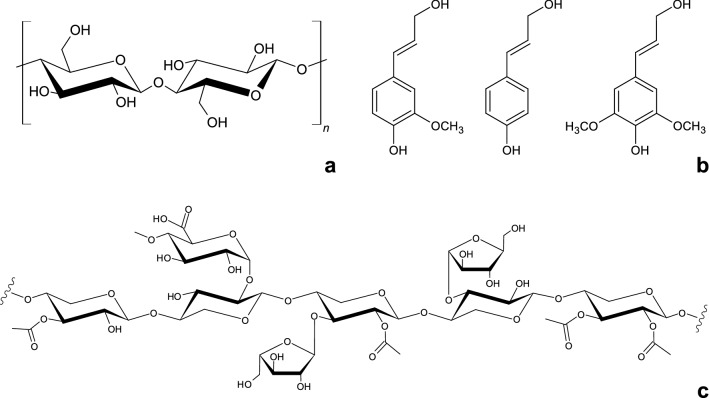
Components of lignocellulose. Chemical structures of cellulose (**a**), the lignin building blocks coniferyl alcohol, *p*-coumaryl alcohol, and sinapyl alcohol (**b**) as well as hemicellulose from grass (**c**) adapted from Escobar et al. (2020)

Probably one of the most important parameters of biomass resources is their composition of cellulose, hemicellulose, and lignin. Together, those three polymers form lignocellulose, which is the major component of plant biomass (Pérez et al. 2002). While cellulose and hemicellulose are macromolecules composed of sugars, lignin is an aromatic polymer, which is synthesized from phenylpropanoid precursors (Pérez et al. 2002). Cellulose is a linear polymer composed of subunits of *D*-glucose, which are linked by ß-1,4-glycosidic bonds (Pérez et al. 2002; Peterson et al. 2008). The so-called cellobiose molecules form fibrils, which are linked together by hydrogen bonds as well as van der Waals forces (Pérez et al. 2002). Due to these bonds, the monomers are crystalline as well as resistant to swelling in water and enzymatic attacks (Peterson et al. 2008). But if water with high temperature and pressure is applied, it can break the hydrogen-bound crystalline structure and hydrolyze the ß-1,4-glycosidic bond, which results in glucose monomers (Peterson et al. 2008). Hemicellulose, on the other hand, does not contain as many repeating ß-1,4-glycosidic bonds and has a more random structure, resulting in a less crystalline and less resistant structure than cellulose (Peterson et al. 2008). Hemicellulose is a polysaccharide comprising *D*-xylose, *D*-mannose, *D*-galactose, *D*-glucose, *L*-arabinose, 4-O-methyl-glucuronic acid, *D*-galacturonic acid, and *D*-glucuronic acid, which are not only linked by ß-1,4-glycosidic bonds but also by ß-1,3-glycosidic bonds (Pérez et al. 2002). Therefore, hemicellulose contains branches and is more vulnerable to hydrothermal extraction or hydrolysis than cellulose (Pérez et al. 2002; Peterson et al. 2008). Lignin is an amorphous heteropolymer consisting of phenylpropane units, which are linked by multiple different bonds (Pérez et al. 2002). It consists of the monomers coniferyl alcohol, sinapyl alcohol, and *p-*coumaryl alcohol, while the relative composition varies strongly depending on the source. Lignin from grass comprises mainly guaiacyl and syringyl units and additionally *p-*coumaric acid and ferulic acid (Lin 1992). Within the cell wall, lignin acts as structural support and a permeability barrier as well as resistance against microbial attacks and oxidative stress (Pérez et al. 2002). The structures of cellulose, typical grass hemicellulose, and lignin monomers are shown in Fig. 1.

The composition of the lignocellulose components varies considerably between different plants. The cellulose/lignin ratio of herbaceous materials like grass is much higher than the ratio of woody materials like branches (Komilis and Ham 2003). Non-woody biomass has a lignin content of around 5–25% (Bagby et al. 1971; Barakat et al. 2013). The amount differs even within species and depends among others on the maturity of the plant (Sawatdeenarunat et al. 2015). Lawn grass for example contains high cellulose (41.7%), high hemicellulose (35.8%), and low lignin content (8.0%) (Guo et al. 2015). Cellulose, hemicellulose, and lignin content of grassland from Oregon, USA ranged from 28.8 to 36.0%, 18.4 to 24.7%, and 13.4 to 17.4%, respectively (Juneja et al. 2011). Premjet et al. showed the composition of 77 indigenous weed species from Thailand varying from 16.1 to 56.0% in cellulose, from 3.3 to 34.2% in hemicellulose, and from 4.6 to 20.4% in lignin (Premjet et al. 2012). Leaves often contain a lower amount of cellulose and hemicellulose and a higher concentration of lignin compared to grassy material (Komilis and Ham 2003). Jung et al*.* compared the content of cellulose, hemicellulose, and lignin of stems and leaves from *Miscanthus*, switchgrass, reed, and sorghum (Jung et al. 2015). The results showed that the woodier reed (*Phragmites australis*) contains significantly more lignin than *Miscanthus*, switchgrass, or sorghum (Jung et al. 2015). Typically, stalks contain higher concentrations of lignin, but also higher concentrations of cellulose and hemicellulose, than leaves (Jung et al. 2015). At the same time, leaves contain a higher amount of extractives (Jung et al. 2015). There are also leaves from e. g. bamboo (27.7%) or the Java plum (27.4%), which can contain more than 25% lignin (Das et al. 2013). However, pruning from the olive tree has been shown to contain 39.4% cellulose, 16.1% hemicellulose, and only 16.8% lignin (Raspolli Galletti et al. 2012). In Table 1, an overview of the composition of various low-lignin plants with lignin contents less than 25% is shown. This overview demonstrates the variability in the composition of lignocellulose depending on the species and the origin of the plant. However, these differences are additionally related to the mentioned parameters cultivation conditions and maturity during harvest. Besides individual plants or grassland, there have also been some examinations of the lignocellulosic composition of green waste. Zhang and Sun determined a cellulose and hemicellulose concentration of green waste of 25.3 and 46.3%, respectively (Zhang and Sun 2014). According to Komilis and Ham, yard waste contains around 27% cellulose, 11% hemicellulose, and 24% lignin (Komilis and Ham 2003). The high amount of lignin, in this case, arises due to a high amount of woody branches in the yard waste.

**Table 1:** Cellulose, hemicellulose, and lignin content from various low-lignin plants

Plant	Origin	Cellulose [%]	Hemicellulose [%]	Lignin [%]	References
Alfalfa (*Medicago sativa*)	Québec, Canada	27.4	11.7	4.8	Alvo and Belkacemi (1997)
Asoka leaves (*Saraca indica*)	India	26.6	30.1	21.8	Das et al. (2013)
Dwarf Napier grass (*Pennisetum purpureum cv. Mahasarakham*)	Thailand	35.6	34.2	3.7	Wongwatanapaiboon et al. (2012)
Eucalyptus leaves (*Eucalyptus marginata*)	India	35.7	47.4	16.9	Das et al. (2013)
Finger flatsedge (*Cyperus digitatus*)	Malaysia	44.8	42.8	11.8	Emmclan et al. (2018)
Greater club rush (*Scirpus grossus*)	Malaysia	36.2	49.9	13.4	Bidin et al. (2015)
	Malaysia	42.1	45.6	11.4	Emmclan et al. (2018)
King Napier grass (*Pennisetum Purpureum x P. Americanum*)	Thailand	32.0	31.1	3.1	Wongwatanapaiboon et al. (2012)
Lesser bulrush (*Typha angustifolia*)	Malaysia	44.1	54.8	20.0	Bidin et al. (2015)
Mango leaves (*Mangifera indica*)	India	27.2	54.0	18.9	Das et al. (2013)
*Miscanthus sacchariflorus*	Korea	31.7–36.1	22.3–28.9	14.1–18.1	Jung et al. (2015)
*Miscanthus sinensis*	India	32.4	21.3	19.0	Jung et al. (2015)
	Korea	30.5–33.4	24.6–29.9	14.7–24.7	Jung et al. (2015)
*Miscanthus* × *giganteus*	Illinois, USA	33.2	23.3	17.2	Jung et al. (2015)
Mission grass (*Pennisetum polystachion*)	Thailand	40.0	23.3	6.2	Premjet et al. (2012)
	Thailand	39.8	29.2	14.6	Tatijarern et al. (2013)
Napier grass/elephant grass (*Pennisetum purpureum*)	Brazil	36	22	20	Menegol et al. (2016)
	Thailand	32.9	36.5	3.6	Wongwatanapaiboon et al. (2012)
Neem leaves (*Azadirachta indica*)	India	20.6	50.8	18.5	Das et al. (2013)
Pangola grass (*Digitaria eriantha*)	Thailand	33.1	35.5	4.5	Wongwatanapaiboon et al. (2012)
Poplar leaves (*Populus nigra*)	India	29.4	48.8	21.8	Das et al. (2013)
Purple guinea grass (*Panicum maximum*)	Thailand	33.4	31.3	4.0	Wongwatanapaiboon et al. (2012)
Purple nutsedge (*Cyperus rotundus*)	Malaysia	42.6	45.6	9.5	Bidin et al. (2015)
Rice flatsedge (*Cyperus iria*)	Malaysia	44.6	43.4	10.6	Emmclan et al. (2018)
	Thailand	33.4	31.0	6.3	Premjet et al. (2012)
Ruzi grass (*Brachiaria ruziziensis*)	Thailand	33.6	34.0	4.6	Wongwatanapaiboon et al. (2012)
Sorghum (*Sorghum bicolor* (L.) Moench)	Nigeria	31.4	23.4	17.9	Jung et al. (2015)
	Sudan	35.3	23.6	20.7	Jung et al. (2015)
Sorghum (*Sorghum halepense*)	Thailand	44.4	25.8	6.6	Premjet et al. (2012)
Switchgrass (*Panicum virgatum*)	Illinois, USA	29.5–37.8	21.5–27.4	13.9–21.1	Jung et al. (2015)
	USA	35.3	33.7	8.4	Reza et al. (2013)
Tall fescue grass (*Festuca arundinacea Schreb*)	Oregon, USA	31.0	20.2	14.4	Kumar and Murthy (2011)
Timothy (*Phleum pratense*)	Québec, Canada	28.8	27.2	4.6	Alvo and Belkacemi (1997)
Ubon paspalum (*Paspalum atratum*)	Thailand	34.9	32.6	5.6	Wongwatanapaiboon et al. (2012)
Vetiver grasses (*Chrysopogon zizanioides*)	Thailand	31.9–38.5	37.9–42.6	3.7–5.1	Wongwatanapaiboon et al. (2012)
Water hyacinth (*Eichhornia crassipes*)	Indonesia	43.0	29.1	6.9	Asrofi et al. (2018)
	Thailand	57.0	25.6	4.1	Tanpichai et al. (2019)
Wild grass (*Achnatherum hymenoides*)	India	51.2	30.1	18.7	Das et al. (2013)

## Pretreatment methods of lignocellulosic biomass

**Fig. 2:**
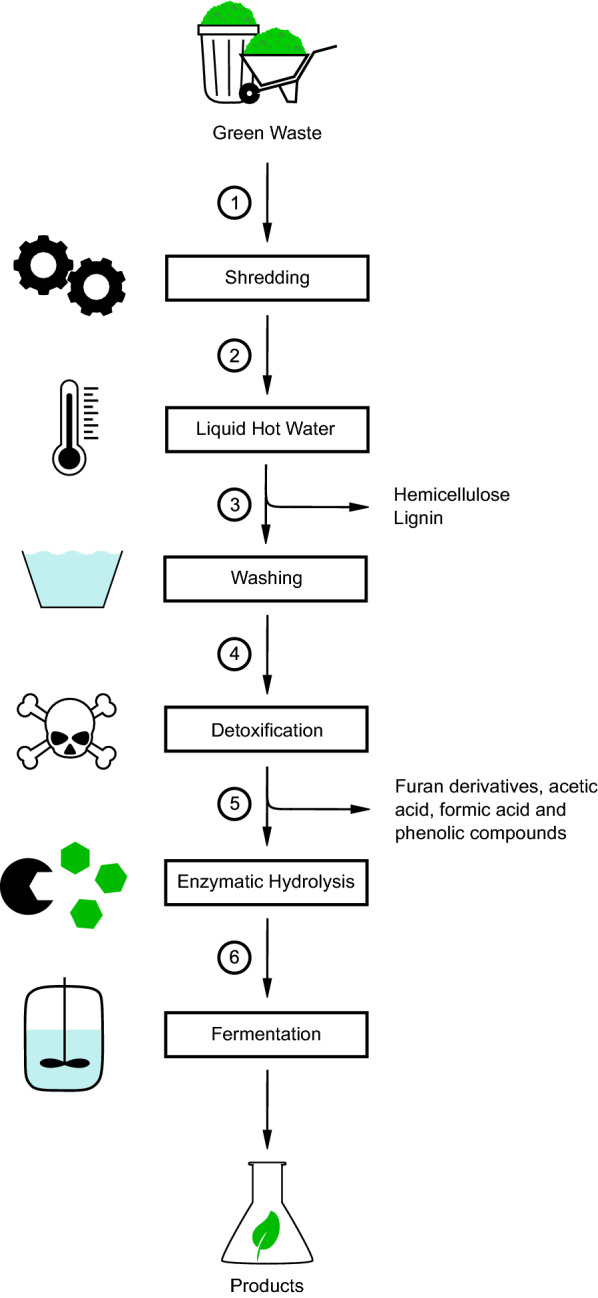
Exemplary pretreatment process of green waste for fermentative utilization. Green waste is shredded (1) and pretreated using elevated temperature and pressure, removing hemicellulose and lignin (2). The resulting hydrolysate is washed (3) and detoxified, e.g., using activated carbon, to eliminate furan derivatives, acids, and phenolic compounds (4). The process is followed by an enzymatic hydrolysis to obtain monosaccharides (5), which are then used in microbial fermentations (6)

As described above, lignocellulosic biomass contains valuable components, which makes it an interesting feedstock for industrial applications. Constituting up to 60% of total biomass (Álvarez et al. 2016), one of the main components of interest are carbohydrates, which can serve as a carbon source for fermentation. The conversion of biomass usually starts with size reduction, followed by one or several pretreatment steps. Subsequently, monomeric sugars like glucose and xylose are obtained via hydrolysis, which can then be converted by various microorganisms into desired products like bulk and fine chemicals. An exemplary pretreatment process of green waste for fermentative utilization is given in Fig. 2. Factors impacting the digestibility of biomass include the chemical composition, notably the lignin, ash, acetate, hemicellulose, and cellulose content as well as the particle size, surface area, pore volume, and the crystallinity of the cellulose (Chang and Holtzapple 2000). Therefore, the pretreatment is crucial for further conversion of the biomass. The main goals of the pretreatment are delignification and structural conversion of cellulose and hemicellulose, making them more accessible for the ensuing hydrolysis into monomeric sugars by enzymes. Yang and Wyman summarized the main requirements for a successful pretreatment procedure of lignocellulosic biomass. The process should result in a liquid hydrolysate with a high concentration of fermentable sugars compatible with the fermenting microorganisms. Occurring by-products should be recovered for further use as far as possible. To reduce costs, the pretreatment method should require as little heat and power as possible, expensive and possible hazardous chemicals should also be avoided (Yang and Wyman 2008). The impact of a successful pretreatment method is emphasized for example by the findings of Rezende et al. By optimizing the pretreatment strategy the sugar release from elephant grass leaves was increased fivefold compared to a sample without pretreatment, resulting in a yield of 205 mg of reducing sugars per gram substrate via acid–alkali pretreatment (Rezende et al. 2018).

Because of the great importance of pretreating lignocellulosic biomass prior to fermentation, there exists a broad variety of different methods. Hendriks and Zeeman identified the increase of the accessible surface area as the critical factor, which is present in all pretreatment methods (Hendriks and Zeeman 2009). According to their underlying principle, the methods usually are subdivided into physical, chemical, physicochemical, and biological methods, although most procedures combine several principles. In this chapter, a short overview of different methods and their underlying principles is provided. Focussing on the pretreatment of green waste, insights stemming from the pretreatment of other lignocellulosic biomass feedstocks like agricultural waste are mentioned in case the principle is applicable to low-lignin green waste biomass. Table 2 summarizes the mentioned methods together with their respective effects. For more detailed and in-depth reviews with a broad range of substrates see for example the publications of Kucharska et al. (2018), Mohapatra et al. (2017), Hendriks and Zeeman (2009), Yang and Wyman (2008), and Taherzadeh and Karimi (2008).

**Table 2:** Overview of pretreatment methods for lignocellulosic biomass

	Size reduction	Fractionation	Reduction of cellulose crystallinity	Dissolution of lignin	Dissolution/hydrolysis of hemicellulose	Dissolution/hydrolysis of cellulose	Delignification	Removal of aromatic compounds	References
Physical									
Milling, etc.	+	+							Holtzapple et al. (1991), Licari et al. (2016)
Irradiation			+						Jusri et al. (2018)
Chemical									
Acidic pretreatment				+	+	+			Kucharska et al. (2018),
Alkaline pretreatment			+						Rabemanolontsoa and Saka (2016), Xu et al. (2010)
Organic solvents					+	+	+		Kucharska et al. (2018), Schmetz et al. (2019)
Ionic liquids, DES						+	+		Remsing et al. (2006), Yu et al. (2019)
Oxidation processes				+	+	+			Hendriks and Zeeman (2009), M'Arimi et al. (2020)
Physicochemical									
Liquid Hot Water					+				Kumar et al. (2009), Yu et al. (2016)
Steam explosion		+		+	+	+			Kim (2018)
Supercritical water		+							Akalın et al. (2017), Peterson et al. (2008)
AFEX			+						Holtzapple et al. (1991), Mes-Hartree et al. (1988)
ARP					+		+		Mes-Hartree et al. (1988), Yoon et al. (1991)
Ultrasound				+	+	+			Bussemaker et al. (2013)
Biological									
Wood-rot fungi							+	+	Akin et al. (1995), Dionisi et al. (2015), Kerem et al. (1992), Sun and Cheng, (2002)
Enzymes					+	+	+	+	Bayer et al. (2004), Gutiérrez et al. (2012), Prawitwong et al. (2013), Rabemanolontsoa and Saka (2016)

### Physical pretreatment methods

Physical pretreatment mainly focuses on the size reduction of biomass. Because of the resulting increase in the specific surface, enzymatic hydrolysis is more effective (Dasari and Berson 2007). Therefore, fragmentation methods like cutting, shredding, grinding, and milling are employed. Licari et al*.* compared different milling methods considering their efficiency for later enzymatic hydrolysis as well as their energy consumption. Vibro-ball milled bagasse samples displayed a cellulose conversion rate of 95% in subsequent enzymatic hydrolysis, while ball and centrifugal mills only achieved rates of 76 and 54%, respectively (Licari et al. 2016). As no additional compounds are needed, this approach has a smaller environmental impact than chemical pretreatment methods. A severe disadvantage however is the high energy consumption compared to most chemical pretreatment methods (Holtzapple et al. 1991; Licari et al. 2016).

Other physical pretreatment methods aim at reducing the crystallinity of cellulose to improve accessibility for further conversion. One example is the irradiation with electron beams with doses of up to 1000 kGy, resulting in a 14% reduction of the crystallinity of microcrystalline cellulose (Jusri et al. 2018). Further types of radiation are employed to treat lignocellulosic biomass prior to fermentation. As they are usually coupled with chemical methods, they are grouped into the physicochemical section below.

### Chemical pretreatment methods

Chemical pretreatment methods are based on chemical reactions taking place in aqueous solution between lignocellulosic biomass and various chemical compounds. Most common are treatments with acid and alkaline solutions.

Acid pretreatment aims at breaking up the lignin structure as well as dissolving hemicellulose and depolymerizing cellulose. It takes place at acid concentrations of 10 – 30%, elevated pressures and temperatures, and reaches hemicellulose degradation rates of up to 90% (Kucharska et al. 2018). Here, a balance needs to be found between effective biomass pretreatment and the limitation of inhibitor formation under harsher conditions. The separation in a first stage under mild conditions to dissolve hemicellulose and a second step in a harsher environment to break down cellulose into monomers helps to reduce the occurrence of interfering degradation products. Another approach of acid pretreatment is the dilute acid method. By applying acid concentrations between 1 and 2%, Karapatsia et al*.* achieved hemicellulose conversion rates of up to 81% from the perennial grass *Phalaris aquatica* (Karapatsia et al. 2017).

Alkaline pretreatment is based on the saponification process. The presence of usually ammonia, as well as sodium or calcium hydroxides (Rabemanolontsoa and Saka 2016), causes swelling of the biomass. Thereby, the reduction of cellulose crystallinity results in an increase in the specific surface. Xu et al*.* achieved a 3.8-fold increase in total reducing sugars from switchgrass by applying mild conditions of 50 °C and 1% NaOH for 12 h, resulting in a yield of total reducing sugars of 45% and a delignification rate of 78%. Harsher conditions resulted in a higher degree of delignification of up to 86% but did not lead to an increased yield of fermentable sugars (Xu et al. 2010).

A variety of organic solvents are used to pretreat lignocellulosic biomass as well. Here, the high degree of delignification and hydrolysis of hemicellulose is the result of the hydrolysis of microfibrillar structures by breaking internal lignin and hemicellulose bonds as well as the bonds between the two structures (Kucharska et al. 2018). Schmetz et al*.* describe an increase in delignification of the tall fescue grass *Festuca arundinacea* Schreb. from 23 to 87% using a butanol pretreatment compared to dilute acid. While the effect on the recovery of xylose and glucose is marginal, the use of butanol during pretreatment results in 74% conversion during subsequent enzymatic hydrolysis, compared to 20% following dilute acid pretreatment (Schmetz et al. 2019). A major disadvantage of solvent pretreatment is the necessity to remove the solvent prior to microbial fermentation.

Ionic liquids, salts with a melting point below 100 °C, are applied to dissolve cellulose by reacting with carbohydrate hydroxyl protons and thereby facilitating the hydrolysis into fermentable sugars (Remsing et al. 2006). A variation is the use of deep eutectic solvents (DES). Although having similar properties, DES are different from ionic liquids as they are composed of a eutectic mixture of Lewis or Brønsted acids and bases, while ionic liquids contain only one type of discrete anion and cation (Smith et al. 2014). Yu et al*.* applied a mixture of water and chloroform in a Liquid Hot Water experiment (described below), proposing the formation of an in situ poly-hydrogen bonding DES. This is supposed to selectively delignify biomass without degrading carbohydrate structures. For *Roystonea regia* leaf sheaths delignification rates of up to 59.6% were achieved while glucan and xylan removal rates were 4.8 and 10.8%, respectively (Yu et al. 2019).

M’Arimi et al*.* give an extensive overview of a variety of advanced oxidation processes applied for biomass pretreatment, for example, ozonation, Fenton oxidation, cavitation methods causing the formation of hydroxyl ions, and photocatalysis (M'Arimi et al. 2020). The application of peroxides effects electrophilic substitution, shifts of side chains, and the breaking of aryl–alkyl bonds, which lead to the dissolution of lignin, hemicellulose, and amorphous cellulose (Hendriks and Zeeman 2009; Kucharska et al. 2018).

### Physicochemical pretreatment methods

Physicochemical pretreatment methods combine the usage of chemicals with the application of physical forces. The basis of hydrothermal methods like Liquid Hot Water, steam explosion, and supercritical water is the simultaneous application of high temperature and pressure in aqueous solution. The latter method is reviewed extensively by Peterson et al. (Peterson et al. 2008). The fractionation of biomass is caused by ionic parts of the liquid, where water as well as carbon dioxide is used (Akalın et al. 2017; Kucharska et al. 2018). In the autocatalytic process of steam explosion, acetyl residues are released from the biomass, whereby the produced acetic acid acts as a catalyst for further hydrolysis. The sudden release of pressure is the key element of this method. Although it is cost-effective, the incomplete lignin removal and the formation of inhibitors are major drawbacks (Kim 2018). A similar process is the Ammonia Fiber Expansion (AFEX), where an anhydrous ammonia suspension serves as solvent. It effects a decrease in cellulose crystallinity and an increased accessible surface area (Holtzapple et al. 1991). In the related Ammonia Recovery Process (ARP), ammonia aqueous solution is repeatedly pumped through lignocellulosic biomass at temperatures between 150 and 190 °C, effecting delignification as well as solubilization of hemicellulose (Yoon et al. 1991). Mes-Hartree et al*.* compared the methods steam explosion and AFEX on several substrates, but could not find a superior method suitable for all biomasses (Mes-Hartree et al. 1988). This emphasizes the diversity of combinations of substrates and pretreatment methods. Rollin et al*.* compared the digestibility of switchgrass after cellulose solvent- and organic solvent-based lignocellulose fractionation (COSLIF) and soaking in aqueous ammonia (SAA). They observed a 16-fold increase in cellulose accessibility to cellulase after COSLIF pretreatment, which primarily breaks lignocellulosic bonds, compared to a 1.4-fold cellulose accessibility increase after SAA pretreatment, mainly eliminating lignin. Therefore, they concluded the accessibility of cellulose to cellulases to be more important than the removal of lignin (Rollin et al. 2011).

During Liquid Hot Water treatment, elevated temperature and pressure cause the hydrolysis of hemicellulose and, at temperatures above 180 °C, lignin, leaving cellulose behind (Kucharska et al. 2018; Kumar et al. 2009). By applying 180 °C for up to 60 min, Yu et al. obtained a total xylose yield of 83.6% of the theoretical maximum from switchgrass. The pretreatment enhanced subsequent enzymatic hydrolysis by 113% and thereby nearly achieved the total theoretical digestibility (Yu et al. 2016).

Microwave irradiation is a way of heating the biomass, resulting in the disruption of lignocellulosic structures. It is usually coupled with another, often chemical, method (Chaturvedi and Verma 2013). There is no explicit proof of a higher saccharification rate in subsequent hydrolysis compared to other thermal pretreatments (Li et al. 2016). The mechano-acoustic effect of ultrasound radiation is shown to cause a reduction in lignin condensation at lower frequencies than 40 kHz and an increase in the solubilization of carbohydrates at a higher frequency than 995 kHz (Bussemaker et al. 2013).

### Biological pretreatment methods

Another approach to treat lignocellulosic biomass are biological methods through the application of microorganisms and isolated enzymes. According to their purpose, these methods can be divided into delignification and saccharification. In addition, lactic acid bacteria can be used to pretreat especially herbaceous biomass through ensiling. While the process requires at least two weeks, the produced organic acids preserve the feedstock, circumventing the problem of only seasonally available biomass. Rabemanolontsoa and Saka summarize various microorganisms including a variety of bacteria and fungi applied for biological pretreatment (Rabemanolontsoa and Saka 2016).

Biological delignification is mainly based on fungi and fungal enzymes. Wood-rot fungi, more specific white-, brown-, and soft-rot fungi, are known to degrade cellulose and lignin (Kerem et al. 1992; Sun and Cheng 2002). Akin et al*.* examined the delignification of Bermuda grass stems by white-rot fungi *Ceriporiopsis subvermispora* and *Cyathus stercoreus*. They showed removal of up to 41% of total aromatic compounds, mainly *p-*coumaric and ferulic acid, while significantly improving the biodegradability through ruminal microorganisms by up to 77% (Akin et al. 1995). Dionisi et al*.* showed a lignin degradation rate of up to 83% by mixed cultures. This applied especially for biomass with a low initial lignin content of 6–10% (Dionisi et al. 2015). One major disadvantage of biological delignification is the processing time. Dionisi et al*.* calculated lignin degradation rates for the microbial delignification process and compared them with common, non-biological approaches as described in Wyman et al. (Wyman et al. 2011). Biomass like cotton stalk had delignification rates of up to 0.1 g L^−1^ h^−1^. Compared to up to 23 g L^−1^ h^−1^ for Liquid Hot Water pretreatment, the biological method cannot compete (Dionisi et al. 2015).

The application of lignin-degrading enzymes promises faster results than using whole organisms. One example is the multicopper oxidase laccase (EC 1.10.3.2). It oxidizes substituted phenols and therefore degrades lignin by modifying its three phenolic subunits *p*-coumaryl alcohol, coniferyl alcohol, and sinapyl alcohol. Gutiérrez et al*.* demonstrated delignification rates of elephant grass and eucalypt of up to 35 and 58% by using laccase in combination with 1-hydroxybenzotriazole as a mediator. The subsequent hydrolysis rate of total reducing sugars increased by 10 and 63%, respectively (Gutiérrez et al. 2012).

One of the best-studied group of organisms for biological saccharification are clostridia. Especially *Clostridium thermocellum* is promising because of its tolerance to high temperatures. To degrade polysaccharides, clostridia possess an intricate extracellular complex of enzymes called cellulosome. It consists of cellulases, xylanases, exo- and endo-glucanases, hemicellulases, chitinases, pectate lyases, and lichenases (Bayer et al. 2004). In contrast, other bacteria and fungi produce free cellulases. A challenging aspect of using microorganisms instead of enzymes for saccharification is to prevent further metabolization of generated glucose to other, unwanted products (Rabemanolontsoa and Saka 2016). An issue concerning the use of enzymes is product inhibition. Prawitwong et al*.* avoided the inhibition of *C. thermocellum* with cellobiose through the addition of a thermostable β-glucosidase. In combination with an alkali pretreatment saccharification of 72% of glucan was achieved (Prawitwong et al. 2013).

Usually, no compounds produced during biological pretreatment procedures are inhibitory to further microbial or enzymatic hydrolysis processes (Sindhu et al. 2016). This poses a major advantage over most physicochemical pretreatment methods as there is no detoxification or purification step necessary. Even more, Du et al*.* observed a positive effect of by-products occurring during pretreatment with the fungus *Irpex lacteus* on the subsequent enzymatic hydrolysis. When the by-products were removed by rinsing the biomass after the biological pretreatment, the maximum reducing sugar yield in subsequent enzymatic hydrolysis decreased by 39%. Additionally, enzymatic hydrolysis of enzymatically pretreated biomass could be enhanced by 32% through the addition of the water extract rich in by-products of biological pretreatment (Du et al. 2011).

### Inhibition and detoxification

Hydrolysis of lignocellulosic biomass does not exclusively result in monomeric sugars. Other components are formed as well, unfortunately, many of which inhibit the subsequent microbial fermentation. Depending on their origin, phenolic compounds, furan derivatives, and weak acids are differentiated (Palmqvist and Hahn-Hägerdal 2000a). Degradation of lignin leads to the formation of phenolic compounds. Due to their hydrophobic structure, they can intercalate into biological membranes, thus destroying their functionality as selective barriers. The furan derivative furfural is formed from the pentose xylose, while 5-hydroxymethylfurfural (HMF) is a degradation product from the hexoses mannose, galactose, and glucose. Furfural has a negative impact on cell growth (Palmqvist and Hahn-Hägerdal 2000a), with high concentration leading to cell death (Palmqvist et al. 1999). Both furfural and HMF can react further to formic acid, while levulinic acid is only formed from HMF. Acetic acid results from the degradation of hemicellulose to monomeric sugars. Those weak acids inhibit cell growth by lowering the intracellular pH, which in turn is answered by the cell by pumping protons out of the cell via the plasma membrane ATPase, using ATP (Palmqvist and Hahn-Hägerdal 2000a).

To alleviate the effects of inhibitory substances there exist a variety of biological, physical, and chemical detoxification methods (Jönsson et al. 2013; Palmqvist and Hahn-Hägerdal 2000b). As described above, the enzyme laccase modifies phenolic compounds. Therefore, it can not only be used for delignification in a pretreatment step but also in a downstream process to remove inhibiting phenolic compounds (Fillat et al. 2017). The same applies to lignin peroxidase. Obtained from the fungus *Trametes versicolor*, Jönsson et al*.* showed an increase in glucose consumption and ethanol production during fermentation with *S. cerevisiae* of up to three times compared to an untreated control for both enzymes (Jönsson et al. 1998). Direct treatment of hydrolysate with the fungus *Trichoderma reesei* also resulted in a tripling of ethanol productivity (Palmqvist et al. 1997). One possibility to eliminate inhibitory acids, furfural and vanillin is evaporation and subsequent dilution of the hydrolysate. Satisfactory results were achieved as well with ethyl acetate or diethyl ether extraction (Palmqvist and Hahn-Hägerdal 2000b; Wilson et al. 1989). Due to its adsorptive properties, activated carbon is also successfully employed for detoxification of hydrolysates. Lee et al*.* achieved removal rates of 42% formic acid, 14% acetic acid, 96% HMF, and 93% furfural with 2.5% activated carbon in one hour. Prolonged reaction time and higher activated carbon loadings resulted in complete removal of HMF and furfural as well as 48% removal of acetic acid and 84% removal of formic acid (Lee et al. 2011). In the so-called overliming process the pH is augmented to over 9 through the addition of Ca(OH)_2_ and subsequently decreased to 5.5. This leads to the destruction of inhibitors in the alkali milieu as well as their precipitation (Martinez et al. 2000; Palmqvist and Hahn-Hägerdal 2000b). The procedure removes mainly HMF and furfural, while the effect on organic acids and phenolic compounds is less pronounced (Martinez et al. 2000). Zhang and Ezeji showed that *Clostridium beijerinckii* is able to reduce the inhibitors furfural, HMF, 4-hydroxybenzaldehyde, and *p-*coumaric acid during fermentation of Liquid Hot Water pretreated *Miscanthus* hydrolysate (Zhang and Ezeji 2014).

### Comparability of pretreatment methods

As demonstrated above, there exists a multitude of different pretreatment methods. But as factors like carbohydrate and lignin content, which strongly impact the pretreatment behavior, varies even within one biomass species, there is no single, optimal procedure. The implications of each pretreatment method need to be considered for every substrate and process anew.

In 2000, the Biomass Refining Consortium for Applied Fundamentals and Innovation (CAFI) made an effort to acquire a comparable database covering various pretreatment methods to obtain sugar from corn stover. The tested methods cover AFEX, ammonia recycle percolation, dilute sulfuric acid, flowthrough of hot water or dilute acid, lime, controlled pH hot water, and sulfur dioxide steam explosion pretreatment. The methods achieved total sugar yields between 86.6% for lime and 96.6% for flowthrough pretreatment of maximum possible values. The monomeric sugar yield however ranged from 60.6% for partial flow to 91.5% for dilute acid pretreatment. In general, the results of the different pretreatment methods are in the same range for corn stover (Elander et al. 2009). The project was expanded in 2004 to include poplar wood and switchgrass, covering the same pretreatment methods. The examination of switchgrass resulted in total sugar yields of 67.3% for SAA (replacing the water and energy-consuming ARP) to up to 90.9% for lime pretreatment. The authors emphasize to not select or exclude one method according to their data, as the experimental conditions were not optimized for the substrates (Wyman 2013).

One approach to prevent excessive laboratory testing for pretreatment methods is the design of predictive models. Chang and Holtzapple establish a link between lignin content as well as cellulose crystallinity and digestibility of biomass (Chang and Holtzapple 2000). Payne and Wolfrum built a model to predict not only the composition of biomass feedstocks but more importantly the yield of soluble carbohydrates resulting from a dilute acid pretreatment followed by enzymatic saccharification. The prediction is based on near-infrared spectral data and partial least squares multivariate data analysis. In contrast to other models, it uses 279 samples from six different feedstock groups instead of a single feedstock (Payne and Wolfrum 2015). Bai et al*.* developed a predictive model for the release of sugar from a feedstock mixture of garden waste of the leguminous tree *Bauhinia blakeana Dunn*, rice straw, and sugarcane bagasse during a microwave-assisted hot water pretreatment process. It allows identifying an optimal mixing ratio of the three feedstocks for a xylose yield of 67.8% (Bai et al. 2020). Hoover et al*.* designed a model to grade different herbaceous biomass feedstocks according to their performance in bioconversions. As defining properties, they identified the content of structural glucan, hemicellulose carbohydrates, acid-soluble, and acid-insoluble lignin as well as ash (Hoover et al. 2019).

Although countless articles are published regarding various pretreatment methods with diverse substrates, it is difficult to compare the different results. There is no standardized evaluation protocol to assess the effectiveness of a pretreatment method. One approach is to state the decrease in hemicelluloses (Rollin et al. 2011), another to measure the amount of total reducing sugars before and after the treatment (Xu et al. 2010). A popular method to rate the effectiveness of the pretreatment is comparing the results of a subsequent enzymatic saccharification. Here, the reported information varies from sugar conversion rates to end product yields. An important aspect due to which the results of different studies are not easy to compare is the fact that the enzymatic methods are rarely optimized to achieve maximum yields, but are merely tools to rank different parameter combinations within one study.

One difficulty concerning the rating of the effectiveness of delignification is the formation of so-called pseudo-lignin (Sannigrahi et al. 2011). During acid and hydrothermal pretreatment, the development of aromatic structures is observed. As they display lignin-like properties, they add to the Klason lignin value without emerging from actual lignin, therefore distorting the results. Pseudo-lignin originates from furfural- and HMF-derived substances for example 3,8-dihydroxy-2-methylchromone and 1,2,4-benzenetriol. That is why it occurs primarily when harsher pretreatment conditions are applied, which leads to increased formation of inhibitors (Sannigrahi et al. 2011; Wan et al. 2019).

Since the 1970s there were efforts to describe the effects of different pretreatment conditions in a comparable way (Chornet and Overend 2017). Overend et al*.* suggested an expression to combine the pretreatment factors time *t* in minutes and temperature *T* in °C (Overend and Chornet 1987). This severity factor *R*_*0*_ is shown in Eq. 1. While a higher severity factor indicates harsher pretreatment conditions, all correlations between the factor and pretreatment results should be considered purely empirical (Chen et al. 2007; Kim et al. 2014):

1 $$R_{0} = t \exp \left\{ {\frac{{\left( {T - 100} \right)}}{\omega }} \right\}.$$

The fitted parameter ω is inversely proportional to the activation energy (Chen et al. 2007) and usually determined to be 14.75. However, Kim et al*.* showed that the influence of the temperature is not adequately expressed by this value for the pretreatment of hardwood chips with the Liquid Hot Water method and adapted the severity factor accordingly (Kim et al. 2014).

An extension of the severity factor was developed by Abatzoglu et al*.* (Abatzoglou et al. 1992) and Chum et al*.* (Chum et al. 1990) as combined severity factor *CS* to include the acid concentration of the pretreatment method as well. Ruiz et al*.* (Ruiz et al. 2017) linked the severity factor R_0_ and the combined severity factor *CS* to the expression in Eq. 2:

2 $${\text{l}} {\text{ogCS = logR}}_{{0}} { - }pH.$$

Ideally, a prediction model would suggest the ideal pretreatment method for a feedstock based on its composition. Due to the low content of lignin, green waste generally requires less harsh pretreatment conditions than other lignocellulosic material (Wolfrum et al. 2013; Xu et al. 2010). Therefore, the use of green waste for biomass conversion is advantageous regarding the consumption of energy and chemicals. But there is the need for a universal comparison of the impact of different pretreatment methods. This includes the degradation rate of lignin, the conversion rate of cellulose and hemicellulose into monosaccharides as well as the formation of substances inhibitory to subsequent enzymatic reactions. The severity factor R_0_ enables a classification of the harshness of pretreatment protocols but offers no indication of the product building rates. Up to now, the best comparative methods are studies like the CAFI project, using uniform experimental conditions. However, there is only limited comparability between different studies, among others due to the lack of a consistent reporting format.

## Products obtained by pressing and extraction

**Fig. 3:**
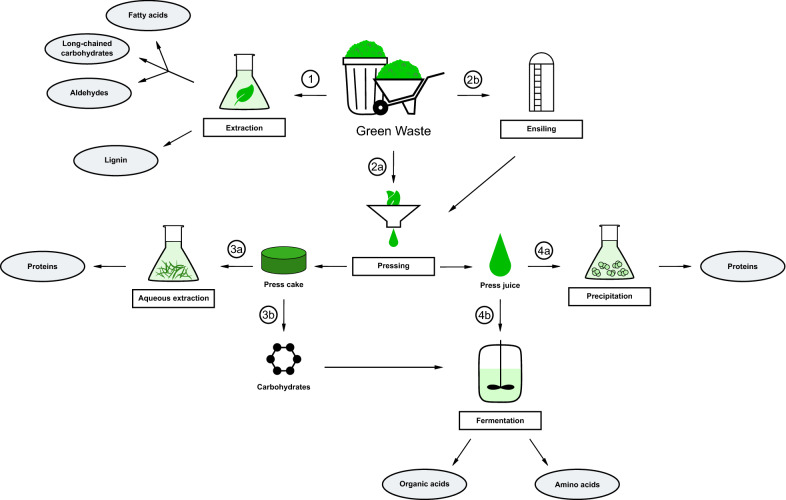
Processes for the production of extracts from green waste. Products are obtained from green waste directly by extraction (1) or via pressing (2a), possibly after ensiling the biomass (2b). After pressing, proteins are obtained from aqueous extraction of the press cake (3a) and precipitation of the press juice (4a). Carbohydrates gained from the press cake (3b) are added to the press juice for fermentation (4b)

A common use of lignocellulosic biomass is the fractionation in lignin, hemicellulose, and cellulose with the subsequent conversion into fermentable sugars. However, green waste contains further valuable compounds. The main products hereof are proteins, organic acids, lipids, and phenolic compounds. Their recovery and utilization are described hereinafter. Figure 3 gives an overview of processes and products from green waste obtained by extraction and pressing.

### Pressing of lignocellulosic biomass

One commonly applied technique for biomass with low lignin content, especially herbaceous material, is its separation into a solid press cake and a liquid press juice by pressing. The press cake usually contains more carbohydrates than the press juice as the cellulosic fibers are less extractable. The press juice however contains a higher amount of crude protein and organic acids. Boakye-Boaten et al*.* obtained a portion of 64% of dry weight cellulose in the press cake of *Miscanthus* versus only 39% in the press juice, while the latter having a protein content of 5% versus only 2.5% in the press cake. Furthermore, 64% of lignin remained in the solid fraction (Boakye-Boaten et al. 2016). As the juice also contains a vast number of organic acids and various nutrients (for a detailed composition of *Miscanthus* press juice see Tables 1 and 2 in Boakye-Boaten et al. 2016), it can serve as a cultivation medium for microorganisms. Boakye-Boaten et al*.* showed improved growth of *Saccharomyces cerevisiae* in 90% *Miscanthus* press juice compared to the commonly used YM medium (Boakye-Boaten et al. 2016).

Andersen et al*.* demonstrated the suitability of press juice from Italian ryegrass (*Lolium multiflorum*), white clover (*Trifolium repens*), and alfalfa (*Medicago sativa*) for the use as a fermentation medium. When glucose was added to reach the same amount as in MRS broth, the growth of lactobacilli in brown juice, press juice depleted of proteins, was higher than in the conventional cultivation medium. It is shown that lactic acid fermented alfalfa juice can replace peptone, yeast, or hydrolyzed soy protein in cultivation media for bacteria while improving growth (Andersen and Kiel 2000). By lactic acid fermentation, it is also possible to preserve the juice for further use, for example, the fermentative production of amino acids or organic acids (Andersen and Kiel 2000).

Leiß et al*.* produced *L-*lysine-*L-*lactate, a precursor for polylactic acid, with alfalfa press juice as a medium for lactobacilli. After the amount of sugar was adjusted to the same level as in the MRS medium, the lactate production rates of 6.4 g L^−1^ h^−1^ for the MRS medium and 6.8 g L^−1^ h^−1^ for press juice were comparable. The deproteinization of the press juice had no significant effect on the lactate production rate, allowing to gain surplus value from the extracted proteins for example as functional food or a foaming agent (Leiß et al. 2010). The suitability of alfalfa press juice for lactic acid production by *Bacillus coagulans* was confirmed by Papendiek and Venus. Again, the supplementation of glucose is necessary (Papendiek and Venus 2014). For an integrated biorefinery process, the additional glucose necessary to use press juice as a fermentation medium could be obtained from the separated press cake via enzymatic saccharification. We already described this process for brewers’ spent grain (Akermann et al. 2019).

### Fermentation of silage press juice

A common approach for the processing of herbaceous biomass is ensiling. As shown above, biomass press juices can serve as cultivation media for fermentation procedures. Several studies examine the suitability of silage press juice for the same application. We obtained a press juice containing 20 g L^−1^ glucose, 17 g L^−1^ xylose/galactose, 44 g L^−1^ fructose/arabinose, and 44 g L^−1^ lactic acid from grass silage based on perennial ryegrass (Sieker et al. 2011). As the acid has a strong inhibitory effect on *S. cerevisiae*, the press juice is not suitable to use as a fermentation medium for the production of ethanol. The usage of hydrolyzed silage press cake also resulted in unsatisfactory yields as nutrients required for fermentation are washed out from the solid fraction. However, they can be replaced by adding up to 10% of the press juice, resulting in a significant increase of productivity compared to the use of a mixture of salts, vitamins, and trace elements and a total ethanol yield of 91 g kg^−1^ (Sieker et al. 2011).

Cerrone et al*.* demonstrated the production of polyhydroxyalkanoates (PHA) by *Burkholderia sacchari* and *Pseudomonas chlororaphis* using press juice from ensiled perennial ryegrass as a carbon source. The juice contained 54 g L^−1^ of total sugars, 20 g L^−1^ of proteins, 15 g L^−1^ of *L*-lactic acid, and 5.4 g L^−1^ of *D*-lactic acid. The acid content is significantly lower than described by Sieker et al*.* (Cerrone et al. 2015). For storage, the press juice was concentrated by rotary evaporation to a content of 300 g L^−1^ total sugar and fermentation was conducted at 20 g L^−1^. With *Burkholderia sacchari*, 44.5 g L^−1^ cell dry weight was achieved, with 33% being polyhydroxybutyrate (PHB), while 37 g L^−1^ cell dry weight of *Pseudomonas chlororaphis* contained 10% PHA (Cerrone et al. 2015). Koller et al*.* examined the addition of green grass juice and silage juice to fermentation media for the production of PHA by *Cupriavidus necator*. Supplementing the minimal medium with 5% (v/v) green grass juice resulted in a productivity of 0.28 g L^−1^ h^−1^ of PHB, which is no improvement compared to the productivity in minimal medium. Supplemented with 5% (v/v) silage juice, however, 0.65 g L^−1^ h^−1^ were achieved, exceeding the result obtained with the common addition of casamino acids by 5% (Koller et al. 2005).

### Extraction of proteins

The extraction of proteins from green waste biomass with low lignin content is studied since 1942 (Pirie 1942). It is proposed to pulp the biomass with added water and then press the material. To precipitate the protein, salt or acid is added to the press juice. On a larger scale, heating the fluid is a more convenient process. Up to 60–70% of the press cakes’ dry matter is protein (Pirie 1969). Dotsenko and Lange examined the recovery of protein from white clover and ryegrass press cake. Using aqueous extraction, 40% of the crude Kjeldahl protein content, which is 10% for white clover and 16% for ryegrass, could be recovered. Treatment with proteases increased the recovery rate to 80% and previous addition of carbohydrases improved the rate further to 95% (Dotsenko and Lange, 2017). LaCour et al*.* increased the amount of precipitated protein in press juices from ryegrass, red clover, a grass–clover mixture, and spinach by adding lignosulfonates from 34–46 to 41–55% (La Cour et al. 2019).

Another method to obtain protein from biomass is the single-cell protein method. Hereby, the biomass serves as a substrate for microorganisms with high protein content. Pihlajaniemi et al*.* use pretreated ensiled grass hydrolysates as media for the fungus *Paecilomyces variotii*, which is known to have a protein content of about 50%. Thereby, the overall protein yield does not only stem from the direct extraction of the biomass, but rather from the nutrients metabolized by the fungus. By using an ammonia-based treatment, part of the residual nitrogen can also be incorporated in the produced proteins (Pihlajaniemi et al. 2020).

### Extraction of lipids and acids

Other value-added products obtainable from green waste biomass as feedstock are lipids or acids. However, there are only a few publications on their extraction. For example, Attard et al*.* extracted various lipophilic compounds from *Miscanthus* pretreated with supercritical carbon dioxide. From the leaves a yield of approximately 2% wax was obtained, containing long-chain hydrocarbons, fatty acids, *n-*policosanols, aldehydes, wax esters, sterols, and steroid ketones (Attard et al.^2016^).

Bichot et al*.* pretreated *Miscanthus* with microwaves and extracted 0.6% ferulic acid and 3.9% coumaric acid (Bichot et al. 2019). According to Karlen et al*.*, extraction titers of at least 50 g kg^−1^ biomass are necessary to make the extraction of hydroxycinnamic acids economically feasible (Karlen et al. 2020).

### Extraction of lignin

Although the lignin share of lignocellulosic biomass is problematic for the production of fermentable carbohydrates, it is also a valuable component of interest itself. Due to its abundance, it is an attractive feedstock to obtain various aromatic compounds. Common industrial extraction methods are Kraft and sulfite processes, both of which apply harsh conditions. This results in the formation of various stable polymers from intermediates, which complicate further use of the lignin components such as Kraft lignin and lignosulfonates (Bertella and Luterbacher 2020). Although the organosolv process is a gentler extraction method, the hereby obtained lignin is highly polymerized as well. Generally, the degree of polymerization can be correlated with the severity of the pretreatment conditions (Dababi et al. 2016). Schwarz et al*.* demonstrated a lignin yield of 41% from organosolv treatment of grass silage (Schwarz et al. 2016).

A possible use of lignin is as a component in bio-polymers (Bertella and Luterbacher 2020). Other conceivable applications are nano-composites, bio-surfactants, and phenolic resins (Alzagameem et al. 2018). Due to its antioxidative and bioactive properties, lignin could also be used as an antimicrobial agent (Alzagameem et al. 2019). Huang et al*.* show further potential target chemicals derived from lignin such as vanillin, polyhydroxyalkanoates, muconic acid, cyclohexanes, and phenols (Huang et al. 2020). Microorganisms with the ability to secrete ligninolytic enzymes and to take up aromatic degradation products of lignin are a promising starting point for a consolidated bioprocess based on lignin (Salvachúa et al. 2015).

The direct extraction of lipophilic compounds such as fatty acids, long-chained carbohydrates and aldehydes from green biomass is not profitable due to the low yields received. The extraction of lignin, often as a by-product during pretreating green waste to obtain fermentable sugars, is more promising. But although there are several interesting approaches to utilize lignin, an industrial process for its valorization is still lacking. However, obtaining products via pressing green waste is seminal. Proteins are successfully extracted as feed supplement. Acidic precipitation of proteins can be conducted chemically by adding for example propionic acid (Brugger et al. 2016) or by fermenting the press juice using lactic acid bacteria (Santamaría-Fernández et al. 2020), reaching crude protein yields of up to 430 g kg^−1^ dry matter. Therefore, green waste is able to compete with usually employed soybean matter (Brugger et al. 2016). The press juice contains a range of nutrients suitable to replace or supplement fermentation media, even after depletion of proteins. The contained sugars are a valuable carbon source for fermentative processes, e.g., the production of PHA. Summarizing, green waste press juice constitutes an alternative for expensive fermentation media compounds.

## Chemical and biotechnological conversion of green waste

**Fig. 4:**
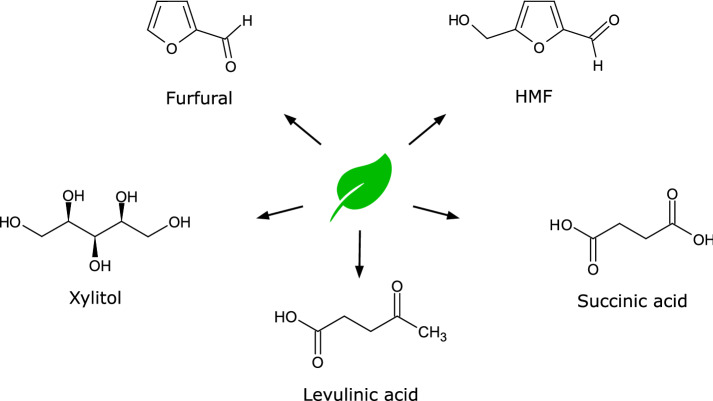
Potential chemicals from herbaceous lignocellulosic materials

The possibility of chemical or biotechnological conversion of biomass components to chemicals has already been shown in large quantities, while the conversion of green waste has not been investigated extensively yet. A relatively simple conversion of green waste, in particular, the included cellulose, hemicellulose, or lignin, to bulk and fine chemicals is a promising way to generate higher value. It is important that green waste is not processed too extensively as the recycling should be financially worthwhile. The U.S. Department of Energy identified 12 possible sugar-derived value-added building block chemicals from biomass (Werpy and Petersen 2004). Succinic acid, levulinic acid, and xylitol are three of these building blocks, which have already been shown to be produced from various herbaceous green materials. Other frequently produced chemicals are furfural and HMF, which have already been described as inhibitors of fermentation but are also valuable basic chemicals. The production of these promising substances as well as other potential candidates by chemical and biotechnological conversion of green waste or herbaceous biomass is described hereinafter. The most promising substances are shown in Fig. 4.

### Production of levulinic and succinic acid

Especially levulinic and succinic acid are popular building block chemicals produced from herbaceous biomass. Levulinic acid is obtained via an acid-catalyzed reaction. Girisuta et al. demonstrated the optimized hydrolysis of water hyacinth to levulinic acid catalyzed by sulphuric acid (Girisuta et al. 2008). The washed, chopped and dried leaves of the water hyacinth were incubated with an aqueous solution of sulphuric acid at constant temperature and various reaction times. The reaction parameters temperature, sulphuric acid concentration, and water hyacinth intake have been investigated. High acid concentrations > 0.5 M yielded levulinic acid as the major organic acid with a maximum yield of 53 mol% based on the amount of C6-sugars. Raspolli Galletti et al. showed the hydrothermal conversion of olive tree pruning to levulinic acid in the presence of homogeneous acid catalysts (Raspolli Galletti et al. 2012). The powdered biomass was incubated with niobium phosphate and water with HCl in an autoclave under an inert atmosphere. At 200 °C, the reaction resulted in up to 66% levulinic acid of the theoretical yield. Furthermore, Raspolli Galletti et al. demonstrated the hydrothermal conversion of giant reed (*Arundo donax* L.) to levulinic acid in the presence of dilute HCl (Raspolli Galletti et al. 2013). The reaction yielded up to 23.3% of levulinic acid based on dry biomass, corresponding to a maximum theoretical yield of 82.7%. A similar process was also shown by Antonetti et al. (2015). They investigated the production of levulinic acid from giant reed in the presence of HCl and were able to achieve a theoretical yield of up to 90%. Dussan et al. demonstrated the acidic hydrolysis of *Miscanthus* × *giganteus* cellulose and hemicellulose with H_2_SO_4_ in a two-stage process into levulinic acid (58–72 mol%) and furfural (27 mol%), respectively (Dussan et al. 2013).

Succinic acid is usually produced through the fermentation of herbaceous biomass. Dąbkowska et al. showed the fermentative production of succinic acid from *Miscanthus* × *giganteus* (Dąbkowska et al. 2019). The biomass was subjected to a glycerol-based pretreatment and enzymatically hydrolyzed. The hydrolysate was fermented with *Actinobacillus succinogenes* 130Z yielding up to 82% succinic acid. Similarly, Kuglarz and Rom showed the succinic acid production from *Miscanthus* × *giganteus* hydrolysate by *A. succinogenes* 130Z resulting in a yield of up to 76% (Kuglarz and Rom 2019). Ventorino et al. demonstrated the succinic acid production from *Arundo donax* hydrolysate with *Basfia succiniciproducens BPP7* (Ventorino et al. 2017). They were able to produce 6.1 g L^−1^ of succinic acid via separate hydrolysis and fermentation in small-scale shake flask experiments. Through optimization of growth conditions and medium composition, a maximal titer of 9.4 g L^−1^ of succinic acid could be obtained with the *A. donax* hydrolysate as well as yeast extract as the main carbon and nitrogen sources in a 2.5 L batch experiment after 24 h. This corresponds to 0.072 kg of succinic acid from 1 kg of pretreated *A. donax* biomass. The highest succinic acid production and yield were achieved with just the liquid fraction. It was assumed that this is due to toxic compounds in the solid fraction of the biomass hydrolysate like e. g. furfural, HMF, *p*-hydroxybenzoic aldehyde, or vanillin. Previously, Ventorino et al. already demonstrated the production of the organic acids lactic acid, succinic acid, and acetic acid from an *A. donax* hydrolysate via the strain *Cosenzaea myxofaciens* BPM1, which was isolated from bovine rumen (Ventorino et al. 2016). Gunnarsson et al*.* demonstrated the conversion of industrial hemp to succinic acid by *Actinobacillus succinogenes* (Gunnarsson et al. 2015). The most effective pretreatment conditions determined were thermochemical treatment with 3% H_2_O_2_ at 121 °C prior to enzymatic hydrolysis, which resulted in a maximum sugar yield of 73.5%. The fermentation with the hemp hydrolysate yielded a maximum of 83% (21.9 g L^−1^) of succinic acid.

### Production of furfural and HMF

The aldehydes furfural and HMF are again produced by chemical processing. Rivas et al. developed a biorefinery strategy for the production of furfural and HMF from *Miscanthus* × *giganteus* (Rivas et al. 2019). *Miscanthus* was pretreated hydrothermally resulting in a soluble hemicellulose-rich and a solid cellulose- and lignin-rich fraction. Acidic processing of the soluble fraction yielded 78% furfural, while enzymatic and subsequent chemical processing of the solid phase resulted in a HMF yield of 49%. Mandalika and Runge demonstrated the sulfuric acid-based dehydration of hot water hydrolyzed *Miscanthus* and switchgrass for the production of furfural with yields of over 90% for both substrates based on the total pentose (Mandalika and Runge 2012). Yang et al. demonstrated the synthesis of furfural and HMF from switchgrass with AlCl_3_·6H_2_O as a catalyst in a biphasic medium of water/tetrahydrofuran (Yang et al. 2012). The reaction system yielded 66% furfural based on the pentose content and 23% HMF based on the hexose content. Zhang et al. demonstrated the dehydration of switchgrass to furfural catalyzed by polymer-bound sulfonic acid in ionic liquid yielding 22% of furfural (Zhang et al. 2014). A conceptual design for the production of furfural and HMF as well as dimethyl furfural from switchgrass has been developed by Martín and Grossmann (Martín and Grossmann 2016). Imteyaz Alam et al. demonstrated the conversion of various weed species (waste plant materials) from India into HMF as well as 5-ethoxymethyl-2-furfural, a promising next-generation biofuel, with a solid acid and ionic liquid catalysts (Imteyaz Alam et al. 2012). Using different Brønsted acidic ionic liquid catalysts resulted in HMF yields ranging from 11 to 58 wt% and 26 to 52 wt% for different weeds, respectively. The direct conversion of the weeds into HMF with a solid acid catalyst yielded 8–32 wt% of HMF. The stated maximum yields were each achieved for foxtail weed. Antonetti et al. showed the hydrothermal conversion of giant reed to furfural in the presence of an acid catalyst as described above. They were able to produce up to 70% of the theoretical yield (Antonetti et al. 2015).

### Production of other valuable substances

The biotechnological production of xylitol from grassy biomass has been shown for certain yeasts, which are able to produce xylitol from the xylose in hydrolyzed biomass. West demonstrated the production of xylitol with the yeast *Candida* on medium containing a hydrolysate of North American perennial prairie grass big bluestem (West 2009). The grass hydrolysate was produced by a combination of dilute acid hydrolysis (1% sulfuric acid) and enzymatic treatment with xylanase (West 2009). Neeru et al. showed the xylitol production from acidic hydrolyzed switchgrass with *Pichia stipitis CBS 5773* (Neeru et al. 2013). They were able to yield 48% xylitol based on the initial xylose.

The fermentative production of polyesters like polyhydroxyalkanoates from grassy biomass is also possible as shown by Davis et al. and Zhang et al. Davis et al. investigated the production of medium-chain length PHA from perennial ryegrass by different *Pseudomonas* strains (Davis et al. 2013). To evaluate different pretreatment methods prior to enzymatic hydrolysis, the grass was pretreated either with 2% NaOH at 120 °C or water alone and with or without a subsequent sodium chlorite/acetic acid treatment for additional lignin removal. The best results were achieved for NaOH and sodium chlorite/acetic acid treatment, resulting in a predominantly glucose-containing hydrolysate. Using the glucose-rich hydrolysates (74–77%) as a sole carbon source resulted in an accumulation of 20–34% PHA of the cell dry mass by the *Pseudomonas* strains, which is comparable to growth and yields with conventional sugars. Zhang et al. demonstrated the production of PHB from alligator weed hydrolysate with *C. necator* (Zhang et al. 2020). The Alligator weed hydrolysate was prepared via acid or enzyme treatment. While the addition of enzymatic hydrolysate as a sole carbon source resulted in an increased PHB production, the acid hydrolysate produced less PHB due to inhibitors affecting microbial growth. After 72 h of fermentation with the enzymatic hydrolysate at optimized conditions, they were able to produce 4.8 g L^−1^ of PHB at a final cell dry weight of 8.5 g L^−1^.

Another example of the production of esters is shown by Cao et al*.* They investigated the chemical cellulose acetate production directly from green landscaping waste after pretreatment with dilute phosphoric acid for separation of hemicellulose (Cao et al. 2018). The pretreated residues were treated with acetic anhydride and sulfuric acid to produce cellulose acetate. The mean yield of cellulose acetate from all green landscaping waste samples was approximately 35%. Xylose and arabinose were obtained as value-added by-products from the pretreatment with dilute phosphoric acid.

The production of fats has also been shown. Employing a mixed culture of the microalga *Chlorella pyrenoidosa* and the yeast *Yarrowia lipolytica* for the fermentation of garden wastes, Yu et al*.* obtained lipid contents of up to 0.8 g L^−1^ (Yu et al. 2019). Mast et al. achieved 0.93 g L^−1^ of mainly long-chain fatty acids by fermenting *Miscanthus* hydrolysates using the red-yeast *Rhodotorula glutinis* over 96 h (Mast et al. 2014).

### Consolidated bioprocessing

When considering the fermentative production of chemicals from green waste, pretreatment e. g. through enzymes and product synthesis by microorganisms are usually separated processes. The combination of enzyme generation, biomass hydrolysis, and the subsequent conversion to final products in a single stage is known as consolidated bioprocessing, which can be achieved by engineering biomass-degrading as well as product synthesis capabilities into one organism (Bokinsky et al. 2011). A dedicated process of enzyme generation for pretreatment of lignocellulose represents a major cost factor, which could be avoided by this approach. Either a synthesis pathway, a biomass degradation pathway or both need to be integrated into the strain without overburdening the organism. For example, Steen et al. were able to engineer a pathway for the production of structurally tailored fatty esters (biodiesel), fatty alcohols, and waxes as well as the expression of hemicellulases into *E. coli* for a possible production of biodiesel from hemicellulose (Steen et al. 2010). A great example of consolidated bioprocessing of green waste has been shown by Bokinsky et al., who engineered biomass-degrading *E. coli*, which are able to grow on cellulose and hemicellulose fractions of plant biomass pretreated with ionic liquids (Bokinsky et al. 2011). They introduced three different biofuel synthesis pathways to produce fuel substitutes or precursors directly from ionic liquid-treated switchgrass. The fermentation on ionic liquid-treated switchgrass as the main carbon source resulted in the production of 71 ± 43 mg L^−1^ fatty acid ethyl esters, 28 ± 5 mg L^−1^ butanol, and 1.7 ± 0.6 mg L^−1^ pinene. Bokinsky et al. furthermore showed that their engineered *E. coli* strains were not only able to grow on pretreated switchgrass but also as mono- and co-culture on pretreated *Eucalyptus globulus* and yard waste. The interesting point in the approach of Bokinsky et al. with regard to green waste processing is that the engineered microorganisms appear to be applicable to a variety of pretreated green biomasses. To fully utilize plant biomass, the combination of microorganisms with both cellulose and hemicellulose degrading abilities could potentially be a promising approach for green waste conversion. Building on that, the desired product synthesis route has to be integrated without overloading the organism to produce value-added chemicals within a consolidated bioprocessing scheme.

## Electrode materials from green waste

**Fig. 5:**
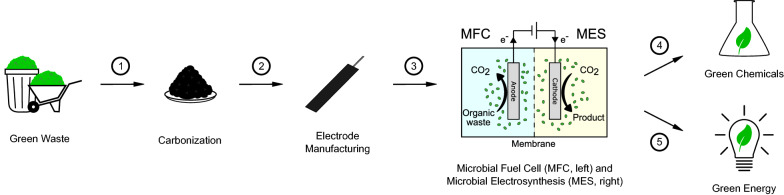
Schematic presentation of electrode manufacturing from green waste and application in bioelectrochemical systems for the production of “green” chemicals or energy: green waste is carbonized and activated through hydrothermal carbonization and/or pyrolysis (1), formed into suitable electrodes (2) and used in electro-biotechnological applications (3) to produce green chemicals in bioelectrosynthesis (4) or green energy in microbial fuel cells (5)

Besides the extraction of valuable compounds and the conversion into basic and fine chemicals, green waste might be utilized profitably as a feedstock for cheap and ecological functional materials. A promising approach to green waste functionalization is its carbonization and subsequent use as electrode material. Carbon-based materials have been proven to be suitable electrodes for electro-biotechnological applications, such as microbial fuel cells or bioelectrosynthesis. Bioelectrochemical systems can contribute to a greener chemistry and bioeconomy. We demonstrated several examples such as bioelectromethanogenesis, allowing conversion of electricity into methane through electroactive methanogens (Enzmann et al. 2019a). The production is influenced among other things by the carbon electrodes used. Besides cathodic working electrodes, we demonstrated that the anodic counter electrode plays an important role in the cathodic bioelectromethanogenesis process as well (Enzmann et al. 2019b). Another example of bioelectrochemical systems are electro-fermentations, which utilize electrical energy to control a reaction in the desired direction as shown for the carbon-efficient production of biobutanol with *Clostridium acetobutylicum* (Engel et al. 2019). High costs due to expensive electrode materials are a major bottleneck of hindering the commercialization of such technologies until now. We already reviewed different types of electrodes, including carbonized materials, for their application in bioelectrochemical systems and highlighted the importance of cost-effective designs (Krieg et al. 2014). Therefore, cheap biomass-derived electrodes could pave the way for commercialization of these novel technologies. The path from green waste to “green” chemicals or energy by functionalization of green waste as electrode material is summarized in Fig. 5.

The carbonization of herbaceous lignocellulosic biomass has already been investigated for the production of coal products as an energy source (Kambo and Dutta 2015, 2014). Especially foliage, which is not suitable for fermentation due to its low content of anaerobically accessible substances, is a promising substrate for carbonization. Coal products from biomass are attractive fuel sources in comparison to raw biomass due to their higher carbon content and calorific value. Additionally, raw biomass causes ignition and combustion problems in combustors due to its high moisture and ash contents (Sadaka et al. 2014). Carbonized biomass is also easy to transport and to store. Biomass-derived carbon materials have already been investigated pretty extensively as electrodes for supercapacitors (Gao et al. 2017; Lu and Zhao 2017).

While biochar production was developed and investigated mainly for woody materials, during the last decades, it has also been studied extensively for herbaceous materials. Carbonized herbaceous biomass is mainly manufactured by hydrothermal carbonization (HTC), producing a solid, so-called biochar, biocoal, or hydrochar (Guo et al. 2015; Medick et al. 2017). During HTC, biomass is carbonized via hot compressed water. While reaction temperatures typically range from 200 to 275 °C, the pressure lies above the saturation pressure of water to ensure its liquidity (Reza et al. 2013). After cooling of the reactor following HTC, the hydrochar is separated by filtration and is dried at 105 °C for 24 h (Eibisch et al. 2013; Guo et al. 2015; Liu et al. 2013; Reza et al. 2013). The structural transformation of cellulose, hemicellulose, and lignin and therefore the characteristics of the biochar depends mainly on temperature and duration as well as the reaction environment (Sadaka et al. 2014). The main products of hydrothermal treatment can be distinguished in solid products (biochar), liquids, and aqueous as well as gaseous (mainly CO_2_), while the properties and distribution of these products depend on the reaction conditions (Liu et al. 2013). Therefore, an important characteristic of HTC is the solid mass yield (Guo et al. 2015). Typically, the HTC of biomass leaves concentrated and dissolved sugars and organic acids in water (Reza et al. 2012). In the sense of a biorefinery concept, it should be considered, whether these dissolved molecules can be further used, although possible unwanted components like HMF are present in the liquid (Yan et al. 2010).

Due to the increasing interest in carbonized materials, there are several publications describing the carbonization of low-lignin materials like grasses, leaves, and even green waste. Reza et al. manufactured and characterized biochar from *Miscanthus* and switchgrass (Reza et al. 2013). The biomass was crushed and dried prior to HTC at 200, 230, or 260 °C with a liquid-to-solid ratio (LTSR) of 1:5 for 5 min. The highest mass yields were achieved at 200 °C resulting in 79 and 87% for *Miscanthus* and switchgrass, respectively. Another carbonization process of switchgrass has been shown by Sadaka et al. (Sadaka et al. 2014). They carbonized ground switchgrass at 300, 350, or 400 °C for 1, 2, or 3 h, but without the addition of water. As described above, biochar mass yield also decreased with increasing temperature and time from 82.6 to 35.2% due to loss of moisture and depolymerization of cellulose, hemicellulose, and lignin. Guo et al. investigated the HTC of lawn grass and the characteristics of the hydrochar (Guo et al. 2015). Lawn grass was dried, shredded, and carbonized at 200 or 240 °C for 30 to 180 min (LTSR 1:30). Solid mass yield ranged from 31 to 50%. A prolonged residence time was shown to be favorable to the formation of hydrochar from lawn grass. Eibisch et al. compared the physicochemical properties of HTC products from various raw materials (Eibisch et al. 2013). The raw materials (woodchips and grass cuttings amongst others) were shredded and hydrochars were produced at 180 °C and 20 bar over 8 h (LTSR 1:10). The hydrochars from grass showed a high amount of hydrophilic functional groups. The higher hydrophilicity of carbonized grass in comparison to hydrochar from woody biomass is advantageous for the use as electrode material since carbonized biomass is already more hydrophobic than the wet feedstock (Acharjee et al. 2011). Liu et al. investigated the HTC of dead eucalyptus leaves under different carbonization conditions (Liu et al. 2013). HTC was performed at 150 – 375 °C for 30 min (LTSR 1:10). Biochar yield decreased rapidly with increasing temperature from a yield of 90% at 150 °C to 30% at 375 °C. Zeymer et al. conducted a technical, economic, and environmental assessment of HTC of green waste for its conversion into coal as an energy source (Zeymer et al. 2017). Prior to the HTC, the green waste was chopped, sieved, and washed. The HTC yielded 76% mass of hydrochar from the green waste. Unfortunately, they did not describe the HTC method further. However, Shao et al. investigated the microwave HTC of green waste consisting of fallen leaves and deadwood (Shao et al. 2019). The green waste was pretreated by removing dirt, milling, and drying at 105 °C for 24 h. They varied the parameters temperature (130–190 °C), holding time (0.5–2 h), and liquid-to-solid ratio (7:1–10:1) with regard to the calorific value of the hydrochar. The yield of hydrochar from green waste ranged from 50.4 to 76.8%.

It has been shown that the biochar mass yield decreases significantly with increasing temperature (Guo et al. 2015; Liu et al. 2013; Reza et al. 2013). Furthermore, carbon content increases with increasing temperature, while oxygen content decreases (Eibisch et al. 2013; Liu et al. 2013). Therefore, the temperature is a major parameter as it influences yield and carbon content. At 200 °C, hemicellulose is eliminated predominantly (Reza et al. 2013) and is almost entirely removed after 30 min (Guo et al. 2015). At reaction temperatures above 200 °C, hemicellulose degrades to the full extent (Reza et al. 2013). The amount of cellulose is also reduced with increasing temperature during HTC (Reza et al. 2013). Cellulose components slowly degrade at 240 °C with increasing residence time to form hydrochar, which is shown by a decrease in the crystallinity index (Guo et al. 2015). While most of the hemicellulose and cellulose already decompose at temperatures below 250 °C, lignin degradation only takes place at higher temperatures around 300 °C (Liu et al. 2013). Due to the loss of cellulose and hemicellulose, lignin percentage increases with reaction temperature (Reza et al. 2013). Reza et al. give a great overview of the composition of hydrochar from grass in comparison to the raw material (Reza et al. 2014). An overview of typically applied process parameters (Temperature, LTSR, pressure, and time) for the HTC of the raw materials described above as well as the resulting biochar mass yield is given in Table 3.

**Table 3:** Investigated parameters for the HTC of herbaceous materials and green waste including hydrochar mass yields

Raw material	Temp. [°C]	LTSR (w/w)	Pressure [bar]	Time	Mass yield [%]	References
*Miscanthus*, switchgrass	200, 230, 260	1:5	10–50	5 min	57–87	Reza et al. (2013)
Lawn grass	200, 240	1:30	N/A	30–180 min	31–50	Guo et al. (2015)
Grass cuttings	180	1:10	20	8 h	N/A	Eibisch et al. (2013)
Eucalyptus leaves	150–375	1:10	N/A	30 min	30–90	Liu et al. (2013)
Green waste	130–190	7:1–10:1	N/A	0.5–2 h	50–77	Shao et al. (2019)

*LTSR* Liquid-to-solid ratio

Although there are several publications on the carbonization of herbaceous materials or green waste, publications addressing the utilization of hydrochars as electrode materials are only sparsely available. However, the usage of other biomass materials as electrodes has been investigated extensively in the last decade. Just recently, Yang and Chen summarized the potential of biomass-derived electrodes for microbial fuel cells (Yang and Chen 2020). They show examples of electrodes from e. g. fruit residues, wood, mushrooms, nutshells, and sludge. The potential of novel electrode materials depends on various parameters like stability, structure, surface area, conductivity, biocompatibility, etc. The pore structure of the electrodes is particularly important for electro-biotechnological applications since the pores have a decisive influence on the bacterial growth on the internal electrode surface as well as substrate and ion transport within the electrode (Yang and Chen 2020). The amount of extractives of biochars from grassy material in comparison to woody material is somewhat higher and therefore indicates a more porous structure than biochar from woody material (Reza et al. 2013). Pore sizes of hundreds of micrometers to millimeters allow bacteria to grow unhindered within the pores, while for smaller pore sizes the biofilm thickness decreases due to limited mass transport within the electrode (Yang and Chen 2020). In addition to the pore size, colonization by bacteria is dependent on surface properties, temperature, and substrate concentration (Yang and Chen 2020). As mentioned previously, hydrochar from grassy material exhibits higher hydrophilicity in comparison to hydrochar from woody biomass (Eibisch et al. 2013), which is advantageous for the growth of microorganisms. Some general advantages of biomass-derived electrodes naturally occurring within biological material are an increased surface area for microbial growth, pores for mass transport of ions and oxygen, high electrical conductivity for fast electron transport, and low resistance as well as the low production costs (Yang and Chen 2020). These characteristics are important prerequisites for their use in commercial and large-scale applications.

Altogether, there are already several publications concerning the carbonization of green waste or herbaceous materials. However, publications investigating the carbonized materials as electrode material in electro-biotechnological applications are lacking. The use of electrodes made of different carbonized herbaceous biomass was shown almost exclusively for the application in capacitors (Jain et al. 2020; Kolanowski et al. 2020; Meng et al. 2020; Saning et al.^2019^). Deng et al. showed the use of carbonized alfalfa leaves, activated with KOH, as a cathodic catalyst in microbial fuel cells (Deng et al. 2017). The biomass-derived carbon material showed superior current density and long-term stability as well as similar performance characteristics in comparison to a Pt/C cathode catalyst. This exceptional example shows that there is still an enormous research potential. Due to promising properties and significantly lower costs compared to conventional electrode materials, electrodes from green waste might be a profitable alternative for electro-biotechnological applications.

## Conclusions

Our overview shows that many valuable chemicals and materials can be produced from green waste. The direct extraction of lipophilic compounds such as fatty acids, long-chained carbohydrates, and aldehydes from green waste does not seem to be profitable due to the low yields received, while the extraction of proteins as well as lignin, which is released as a by-product during pretreatment, is more promising. As potential transformation products of green waste we have identified levulinic acid, furfural, and HMF from chemical conversion and succinic acid, xylitol, and polyhydroxyalkanoates from fermentative conversion. In particular, the fermentative utilization of green waste shows great potential, whether as a carbon source through sugars received after pretreatment or as a supplement in the form of e. g. press juice. In relation to this, a consolidated bioprocess tailored to green waste is a promising perspective. Furthermore, there are several publications concerning the carbonization of green waste materials, whereas the application as electrodes is hardly researched. These “green” electrodes could contribute to novel bioelectrochemical processes in a bio-based economy. However, alternative recycling methods of green waste are far from being as extensively researched as other waste streams. Studies on the material utilization of heterogeneous green waste in its entirety are hardly available. Consequently, economic and ecological evaluations of potential alternatives are also lacking. The comparability of methods for green waste processing from pretreatment to conversion is limited, e. g. there is often a lack of quantitative data regarding energy and material consumption. But the profitable realization of the utilization of green wastes will not depend solely on technical approaches. Collecting and logistics must always be considered as well. For green waste to be recycled economically, it must be done as locally as possible. Nevertheless, the reviewed publications show the untapped potential of green waste. Finally, it is likely that only a cascade-like use of the collected green waste will lead to an economic process. In a first step, the green waste would be pressed. The press juice would be subsequently used for fermentative production of high-value substances. In parallel, electrodes could be produced from the remaining solid biomass by carbonization. After use in electro-biotechnological applications, these electrodes could be utilized energetically, e. g. in biogas plants. This cascade-like processing would result in a green waste-biorefinery.

## Data Availability

Not applicable.

## Abbreviations

AFEX: Ammonia fiber expansion
ARP: Ammonia recovery process
CAFI: Biomass Refining Consortium for Applied Fundamentals and Innovation
COSLIF: Cellulose solvent- and organic solvent-based lignocellulose fractionation
CS: Combined severity factor
DES: Deep eutectic solvents
HMF: 5-Hydroxymethylfurfural
HTC: Hydrothermal carbonization
LTSR: Liquid-to-solid ratio
MSW: Municipal solid waste
PHA: Polyhydroxyalkanoate
PHB: Polyhydroxybutyrate
SAA: Soaking in aqueous ammonia
